# Clinical analysis of microbiologically proven fungal keratitis according to prior topical steroid use: a retrospective study in South Korea

**DOI:** 10.1186/s12886-019-1212-0

**Published:** 2019-10-16

**Authors:** Chan-Ho Cho, Sang-Bumm Lee

**Affiliations:** Department of Ophthalmology, Yeungnam University College of Medicine, 170, Hyunchung-ro, Nam-gu, Daegu, 705-717 (42415) South Korea

**Keywords:** Fungal ocular infection, Steroids, Ulcerative keratitis

## Abstract

**Background:**

To compare the clinical characteristics and treatment outcomes of microbiologically proven fungal keratitis between users and non-users of prior topical steroids (PS and NPS, respectively).

**Methods:**

Eighty-three cases with microbiologically proven fungal keratitis between January 2000 and December 2016 were reviewed retrospectively. Diagnosis of fungal keratitis was made through potassium hydroxide smear, culture, PCR, or biopsy. Baseline epidemiology, predisposing factors, clinical characteristics, microbiological profiles, and treatment outcomes were evaluated and compared between the PS and NPS groups. Treatment failure was defined as any case with complications or requiring surgery. The risk factors for treatment failure were evaluated using multivariate logistic regression in the overall cohort.

**Results:**

A total of 30 cases with PS group and 53 cases with NPS group were included. Of these, sixteen fungal isolates were identified in the PS group and 14 isolates in the NPS group. *Candida* was the most common organism in both groups (6 cases, respectively), while *Aspergillus* (4 cases) was found only in the PS group (*p* = 0.103). No significant differences were observed in the mean age, sex, occupational distribution, epithelial defect size, hypopyon, and presenting best-corrected visual acuity (BCVA) between the two groups. Differences were observed between the PS and NPS groups in terms of previous ocular surface disease (OSD) (43.3% vs. 22.6%, *p* = 0.048) and deep infiltration (53.3% vs. 32.1%, *p* = 0.057). Regarding treatment outcomes, final BCVA < 0.1 (60% vs. 44.2%, *p* = 0.133), the use of voriconazole (topical 10% vs. 0%, *p* = 0.044; systemic 23.3% vs. 1.9%, *p* = 0.003), surgical intervention (43.3% vs. 20.8%, *p* = 0.029), and treatment failure (46.7% vs. 22.6%, *p* = 0.023) were more common in the PS group than in the NPS group. The significant risk factors for treatment failure were hypopyon (odds ratio [OR] 6.01, *p* = 0.005) and deep infiltration (OR 4.38, *p* = 0.013).

**Conclusions:**

Previous OSD and deep infiltration were more common in the PS group compared to the NPS group. The PS group also experienced worse disease progression and treatment outcomes. These results highlight the need for paying attention to the use of steroids in clinical practice.

## Background

Fungal keratitis is an important cause of ocular morbidity and has been reported to account for about half of all microbial keratitis cases requiring therapeutic penetrating keratoplasty [1]. Fungal keratitis is a challenging disease to diagnose and treat. Moreover, it is often confused with other infectious keratitis as there is usually insufficient clinical and microbiological evidence during its early stages thereby leading to delayed treatment. Treatment outcomes for fungal keratitis have been found to be worse compared to bacterial keratitis [2]. Furthermore, there are few commercialized topical antifungal agents, and most of these agents have poor penetration in the cornea [3, 4].

The risk factors for fungal keratitis include ocular trauma, ocular surface disease, contact lens use, topical steroid use, and systemic immunosuppression [5, 6]. Among these, the prior use of topical corticosteroids has been shown to be a clinically important factor because it can exacerbate the infection [7]. The use of topical steroids in the early stages of infectious keratitis makes it difficult to judge clinical progression because the immune-inflammatory response in the corneal stroma is temporarily improved and the immune response of the host is decreased [8].

Regardless, there is limited understanding of the progression and treatment outcomes of fungal keratitis according to prior exposure to topical steroids. Therefore, we conducted a comparative study of patients with microbiologically proven fungal keratitis according to prior topical steroid use at a tertiary referral center in South Korea. The aim of this study was to compare the epidemiology, predisposing factors, clinical characteristics, microbiological profiles, and treatment outcomes in patients with microbiologically proven fungal keratitis according to prior exposure to topical steroids.

## Methods

This study was conducted at the Yeungnam University Hospital, a tertiary referral center in South Korea. We retrospectively reviewed medical records of microbiologically proven fungal keratitis cases between January 2000 and December 2016. The inclusion criteria were clinical evidence of fungal keratitis and clinical response to antifungal treatment plus one of the following: (a) positive fungal culture from a corneal specimen, (b) positive identification of fungal elements on a 10% potassium hydroxide (KOH) smear, and (c) histopathology showing presence of fungal elements. The exclusion criteria were fungal keratitis with scleral involvement and cases did not receive antifungal therapy. For the purpose of this study, the overall cases were divided into two groups: those with prior topical steroid use before the diagnosis of fungal keratitis as PS group, and those with no prior topical steroid use before diagnosis as NPS group. This study was approved by the Institutional Review Board of the Yeungnam University Hospital (IRB No. 2018–11-015), Republic of Korea, and complied with the principles outlined in the Declaration of Helsinki.

Baseline epidemiology, predisposing factors, clinical characteristics, microbiological profiles, and treatment outcomes were evaluated and compared between the PS and NPS groups. The epidemiologic characteristics included age, sex, occupation, symptom duration and whether patient was referred from a primary eye clinic. The symptom duration was defined as the interval from the onset of symptoms to the time of initial presentation. The predisposing factors included previous ocular surface disease (OSD), previous ocular surgery, underlying systemic disease, corneal trauma, and use of contact lens. The initial clinical characteristics included the location, size of the corneal lesion, depth of infiltration, the presence of the hypopyon, and presenting best-corrected visual acuity (BCVA) as determined using the Snellen test. The corneal lesions were divided into central or peripheral lesions based on the half radius of the cornea. The size of corneal lesions was calculated based on the size of the corneal epithelial defect [9]. Depth of infiltration was categorized as either superficial (more than 0 to 50%) or deep (more than 50 to 100%).

Before initiation of therapy, corneal scrapings of all cases were obtained using a No. 15 Bard-Parker knife (Aspen Surgical, Caledonia, MI, USA) after application of 0.5% proparacaine hydrochloride (Alcaine^®^, Alcon, Fort Worth, TX, USA) for anesthesia. Simultaneously, conjunctival swab was performed for all cases using a sterile cotton-tipped swab for thioglycolate broth. Scrapings were smeared on glass slides and Gram staining was performed. For KOH smear, specimen was taken from the margins and base of the ulcer and was placed within a marked area on a glass slide. One drop of 10% KOH was put on it and a clean coverslip was added. The corneal scrapings were stained by the Gram and acid-fast bacilli (AFB) stain methods, and also inoculated onto a variety of solid and liquid media that support the growth of bacteria, fungi, and acanthamoeba. These included thioglycolate broth, blood agar, MacConkey agar, and Sabouraud dextrose agar. Samples were inoculated into Sabouraud dextrose agar for fungal detection and incubated at room temperature or 30 °C for 21 days.

When a fungal infection is clinically suspected or when a fungus is identified, systemic and topical antifungal agent was administered immediately. The first-line antifungal used was topical amphotericin B given hourly. In cases where no response was seen within 72 h, topical 5% natamycin (Natacin^®^, Alcon, Fort Worth, TX, USA) was added to the above regimen. Systemic antifungal agents (fluconazole 50 mg tid p.o. / intravenous amphotericin B) were administered. Topical 1% voriconazole was used in cases where there was no improvement in the lesion even after 2 weeks of continuous use of amphotericin B eyedrop and 5% natamycin, or when anterior chamber fungal ball was formed. All patients were treated topically with 3rd or 4th generation fluoroquinolones and fortified topical antibiotics (2% tobramycin, 5% ceftazidime) and systemic antibiotics before the microbiological results were obtained as empirical treatment. When topical steroids were in use at the initial presentation, they were gradually tapered. The treatment outcomes were assessed at the end of 3 months or at the completion of treatment. Treatment outcomes were evaluated by epithelial healing time (EHT), complication, surgical intervention, and final BCVA. Treatment failure was defined as the occurrence of complications or need for surgical treatment.

The data were analyzed using the Statistical Package for the Social Sciences 20.0 (IBM, Armonk, NY, USA). Chi-square test and Fisher’s exact test were used for categorical data. Independent *t*-tests were used for comparison of mean values. Statistical significance was indicated by *p* < 0.05 (two-tailed). The risk factors for treatment failure were analyzed using logistic regression analysis in the overall cohort. An independent variable with a *p* < 0.1 from the univariate analysis was included in the multivariate analysis and a variable with a final *p* < 0.05 was considered as a significant risk factor.

## Results

### Baseline epidemiology, predisposing factors, and clinical characteristics

Over a 17-year period, we identified 89 microbiologically proven fungal keratitis cases. Of these, six cases had accompanying necrotizing scleritis. These 6 cases were excluded, leaving 83 microbiologically proven cases of fungal keratitis being enrolled in this study. Of these, thirty cases (36.1%) were in the PS group and 53 cases (63.9%) were in the NPS group.

Table 1 compares the baseline epidemiology, predisposing factors, and clinical characteristics of the PS and NPS groups. For the overall cohort, the mean age was 63.0 ± 14.3 years and 57.8% were males. About half of patients were agricultural workers in both groups (PS: 46.7%, NPS: 47.2%). There were no significant differences in the mean age, sex, and occupational distribution between the PS and NPS groups. The median time to symptom duration was 14 days in the PS group and 10 days in the NPS group, the difference being statistically not significant. Among the PS group, 36.7% of topical steroids were used in primary eye clinic and 63.3% were used in our hospital.

**Table 1:** Baseline epidemiology, predisposing factors and clinical characteristics of fungal keratitis according to prior topical steroid use

Characteristics	PS (*n* = 30)	NPS (*n* = 53)	*p*-value
Epidemiology			
Male sex	19 (63.3)	29 (54.7)	0.445
Age, years	60.2 ± 14.9	64.6 ± 13.9	0.180
Occupation			
Agriculture	14 (46.7)	25 (47.2)	0.965
Non-agriculture	16 (53.3)	28 (52.8)	
Symptom duration, days	19.7 ± 15.4	15.0 ± 13.8	0.154
Median (range)	14 (2–150)	10 (1–90)	0.127^†^
Referral from primary eye clinic	11 (36.7)	4 (7.5)	0.001
Predisposing factors			
Corneal trauma	21 (70.0)	43 (81.1)	0.246
Vegetable matter or wood	8 (26.7)	21 (39.6)	0.234
Soil or water	9 (30.0)	16 (30.2)	0.986
Other trauma	4 (13.3)	6 (11.3)	1.000^*^
Previous OSD	13 (43.3)	12 (22.6)	0.048
Herpetic keratitis	7 (23.3)	5 (9.4)	0.108^*^
Punctate keratopathy	3 (10.0)	2 (3.8)	0.346^*^
Old corneal opacity	2 (6.7)	3 (5.7)	1.000^*^
Other keratopathy^a^	1 (3.3)	2 (3.8)	1.000^*^
Contact lens wear	1 (3.3)	4 (7.5)	0.649^*^
Previous ocular surgery	10 (33.3)	10 (18.9)	0.139
Systemic disease	12 (40.0)	21 (39.6)	0.973
Diabetes mellitus	4 (13.3)	10 (18.9)	0.518
Hypertension	7 (23.3)	12 (22.6)	0.943
Initial clinical characteristics			
Central corneal lesion	22 (73.3)	42 (79.2)	0.538
Epithelial defect size (mm^2^)	12.7 ± 12.4	10.5 ± 11.2	0.404
≥ 10 mm^2^	13 (43.3)	16 (30.2)	0.228
Depth of infiltration			
Superficial (0–50%)	14 (46.7)	36 (67.9)	0.057
Deep (50–100%)	16 (53.3)	17 (32.1)	
Hypopyon	9 (30.0)	17 (32.1)	0.845
Presenting BCVA (logMAR)	1.63 ± 0.96	1.47 ± 1.14	0.486
< 0.1, Snellen	20 (66.7)	27 (51.9)	0.194

Values are presented as mean ± standard deviation or number (%)

*BCVA* best corrected visual acuity, *NPS* group of no prior topical steroid use, *OSD* ocular surface disease, *PS* group of prior topical steroid use

^*^The *p*-value was calculated using Fisher’s exact test

^†^The *p*-value was calculated using Kruskal-Wallis test

^a^Include neurotrophic keratopathy (PS), bullous keratopathy (NPS), and exposure keratopathy (NPS)

Corneal trauma (70.0, 81.1%) was the most common predisposing factor in both groups. The ratios of previous OSD (43.3% vs. 22.6%, *p* = 0.048) and previous ocular surgery (33.3% vs. 18.9%, *p* = 0.139) were higher in the PS group. Herpetic keratitis (23.3, 9.4%) was the most common previous OSD in both groups.

Among the initial clinical characteristics, central corneal lesions were more common than peripheral ones in both groups. There were no significant differences in the location of corneal lesions and hypopyon between the two groups. The cases with epithelial defect size ≥10 mm^2^ (*p* = 0.228) and presenting BCVA < 0.1 (*p* = 0.194) were slightly higher in the PS group, but the differences were not statistically significant. There was a higher proportion of cases with deep infiltration in the PS group compared to the NPS group (53.3% vs. 32.1%, *p* = 0.057).

### Microbiological test results

Table 2 shows the microbiological test results including identified fungal isolates and KOH smear result. Thirty fungal isolates out of 30 eyes were identified in the overall group. Of these, sixteen fungal isolates in the PS group and 14 in the NPS group were identified. The most commonly identified fungal organisms were the *Candida* species (6 cases, respectively), followed by *Fusarium* species (3 cases, 5 cases), and *Aspergillus* species (4 cases, 0 case). There was no statistically significant difference between the PS group and the NPS in the distribution of *Candida* (*p* = 0.765) and *Fusarium* species (*p* = 0.417), while the *Aspergillus* species were found only in the PS group (*p* = 0.103). Twenty cases (66.7%) of KOH smear positive in the PS group and 46 cases (86.8%) in the NPS group were identified. Six cases (20%) of both culture and KOH smear positive in the PS group and 7 cases (13.2%) in the NPS group were identified (*p* = 0.532).

**Table 2:** Microbiological test results^a^ according to prior topical steroid use

	PS (*n* = 30)	NPS (*n* = 53)	*p*-value
Identified fungal isolates^b^	16 (53.3)	14 (26.4)	0.014
*Candida* species^#^	6 (37.5)	6 (42.9)	0.765
*Fusarium* species^#^	3 (18.8)	5 (35.7)	0.417^§^
*Aspergillus* species^#^	4 (25.0)	0 (0.0)	0.103^§^
*Syncephalastrum* species^#^	0 (0.0)	1 (7.1)	0.467^§^
*Alternaria* species^#^	1 (6.2)	0 (0.0)	1.000^§^
*Cryptococcus* species^#^	1 (6.2)	0 (0.0)	1.000^§^
*Acremonium* species^#^	1 (6.2)	0 (0.0)	1.000^§^
Unknown species^#^	0 (0.0)	2 (3.8)	0.209^§^
KOH smear positive	20 (66.7)	46 (86.8)	0.029
Identified fungal isolates and KOH smear positive	6 (20.0)	7 (13.2)	0.532^§^

Values are presented as number (%)

*KOH* potassium hydroxide, *NPS* group of no prior topical steroid use, *PCR* polymerase chain reaction, *PS* group of prior topical steroid use

^#^Percentage and *p*-value of each species were calculated based on the identified fungal isolates

^§^The *p*-value was calculated using Fisher’s exact test

^a^Defined as positive result if at least one of the following is included: (a) positive fungal culture from a corneal specimen, (b) positive identification of fungal elements on a 10% KOH smear, (c) positive identification of fungal elements on multiplex PCR, or (d) histopathology showing presence of fungal elements

^b^Identified by culture, multiplex PCR, and biopsy

### Treatment outcomes

Of the overall patient population, sixty-three cases (75.9%) received topical antifungal monotherapy, while 20 cases (24.1%) received a combined antifungal treatment. Combined topical antifungal treatment (33.3% vs. 18.9%, *p* = 0.139) and voriconazole/natamycin combination (10% vs. 0%, *p* = 0.044) were more common in the PS group than in the NPS. For systemic antifungal agents, fluconazole was the most commonly used in both groups (53.3, 67.9%), followed by amphotericin B (26.7, 32.1%). The use of systemic voriconazole was significantly higher in the PS group (23.3% vs. 1.9%, *p* = 0.003).

The median EHT was 27 days in the PS group and 23 days in the NPS group. Sixty percent of the PS group had a final BCVA of < 0.1 compared to 44.2% in the NPS group (Table 3). As a complication, corneal perforation was the most common (8 cases, 7 cases) in both groups, followed by endophthalmitis (2 cases, 1 case). The proportion of corneal perforation was slightly higher in the PS group compared to the NPS group (*p* = 0.126).

**Table 3:** Treatment outcome of fungal keratitis according to prior topical steroid use

Characteristics	PS (*n* = 30)	NPS (*n* = 53)	*p*-value
Medical treatment: topical			
Antifungal agent monotherapy	20 (66.7)	43 (81.1)	0.139
Amphotericin B	8 (26.7)	19 (35.8)	0.391
Natamycin	12 (40.0)	24 (45.3)	0.641
Combined antifungal agents	10 (33.3)	10 (18.9)	0.139
Amphotericin B/ natamycin	7 (23.3)	10 (18.9)	0.628
Voriconazole/ natamycin	3 (10.0)	0 (0.0)	0.044^*^
Medical treatment: systemic^a^			
Terbinafine	5 (16.7)	4 (7.5)	0.273^*^
Itraconazole	1 (3.3)	4 (7.5)	0.649^*^
Fluconazole	16 (53.3)	36 (67.9)	0.187
Amphotericin B	8 (26.7)	17 (32.1)	0.606
Voriconazole	7 (23.3)	1 (1.9)	0.003^*^
Treatment outcome			
Epithelial healing time, days^b^	44.6 ± 50.5	29.6 ± 27.9	0.165
Median (range)	27 (4–190)	23 (3–150)	0.248^†^
Final BCVA^c^ (logMAR)	1.36 ± 1.20	0.92 ± 1.08	0.133
< 0.1, Snellen	18 (60.0)	23 (44.2)	0.169
Complications			
Corneal perforation	8 (26.7)	7 (13.2)	0.126
Endophthalmitis	2 (6.7)	1 (1.9)	0.295^*^
Surgical intervention	13 (43.3)	11 (20.8)	0.029
AMT	9 (30.0)	4 (7.5)	0.011^*^
Evisceration/enucleation	4 (13.3)	7 (13.2)	1.000^*^
Conjunctival flap	6 (20.0)	4 (7.5)	0.157^*^
Penetrating keratoplasty	0 (0.0)	1 (1.9)	1.000^*^
Time to evisceration/enucleation < 1 month^d^	2/4 (50.0)	5/7 (71.4)	0.576
Duration of hospitalization, days^e^	15.6 ± 6.3	13.0 ± 6.0	0.070
Treatment failure^f^	14 (46.7)	12 (22.6)	0.023

Values are presented as mean ± standard deviation or number (%)

*AMT* amniotic membrane transplantation, *BCVA* best corrected visual acuity, logMAR logarithm of the minimal angle of resolution, *NPS* group of no prior topical steroid use, *PS* group of prior topical steroid use

^*^The *p*-value was calculated using Fisher’s exact test

^†^The *p*-value was calculated using Kruskal-Wallis test

^a^Percent do not add to 100% because some cases had combined systemic medications

^b^Total *n* = 71: cases with persistent epithelial defect were excluded. (3 cases in PS, 9 cases in NPS)

^c^The final BCVA was assessed at the end of 3 months or at the completion of treatment

^d^Percentages and statistical values were calculated within the group of underwent evisceration/enucleation

^e^Total *n* = 77: cases of outpatients were excluded (2 cases in PS, 4 cases in NPS)

^f^Defined as the occurrence of complication or surgical intervention

Overall, twenty-four cases (28.9%) required surgical intervention, the proportion being higher in the PS group (43.3% vs. 20.8%, *p* = 0.029). In the PS group, amniotic membrane transplantation was performed in 9 cases, and 6 cases had conjunctival flap. In the NPS group, evisceration/enucleation was performed in 7 cases. The proportion of evisceration/enucleation (13%, respectively) was similar between the two groups. The proportion of treatment failure was significantly higher in the PS group (46.7% vs. 22.6%, *p* = 0.023) (Table 3).

### Risk factors for treatment failure

In a multivariate logistic regression analysis, hypopyon (odds ratio [OR] 6.01, 95% confidence interval [CI] 1.70–21.23, *p* = 0.005) and deep infiltration (OR 4.38, 95% CI 1.37–14.08, *p* = 0.013) were identified as significant risk factors for treatment failure. Prior topical steroid use (OR 2.79, 95% CI 0.85–9.18, *p* = 0.091) and previous OSD (OR 2.82, 95% CI 0.84–9.49, *p* = 0.093) were not significant in multivariate analysis (Table 4).

**Table 4:** Risk factors for treatment failure^a^ in fungal keratitis using univariate and multivariate logistic regression analysis

Variables	Univariate analysis			Multivariate analysis^†^		
	OR	95% CI	*p*-value	OR	95% CI	*p*-value
Female sex	1.27	0.50–3.23	0.620			
Age ≥ 60 years	1.80	0.62–5.21	0.277			
Agricultural occupation	0.48	0.18–1.25	0.130			
Corneal trauma	0.22	0.08–0.66	0.006	-	-	-
Prior topical steroid use	2.99	1.14–7.84	0.026	2.79	0.85–9.18	0.091
Previous OSD	2.90	1.08–7.80	0.035	2.82	0.84–9.49	0.093
Previous herpetic keratitis	2.55	0.74–8.85	0.140			
Previous ocular surgery	1.25	0.43–3.62	0.685			
Diabetes mellitus	2.63	0.81–8.51	0.106			
Symptom duration ≥10 days	1.34	0.52–3.46	0.543			
Central corneal lesion	1.02	0.34–3.06	0.978			
Epithelial defect size ≥10 mm^2^	2.56	0.98–6.70	0.055	-	-	-
Deep infiltration	8.34	2.90–23.96	< 0.001	4.38	1.37–14.08	0.013
Hypopyon	5.70	2.06–15.80	0.001	6.01	1.70–21.23	0.005
Presenting BCVA < 0.1, Snellen	4.85	1.60–14.67	0.005	-	-	-

*BCVA* best corrected visual acuity, *CI* confidence interval, *OR* odds ratio, *OSD* ocular surface disease;

^a^Treatment failure was defined as the occurrence of complication or surgical intervention

^†^Multivariate logistic regression analysis was performed using the backward-conditional method for the factors with a *p*-value < 0.1 in univariate logistic regression analysis

## Discussion

In this study, 36.1% of patients were exposed to topical steroids prior to their diagnosis of fungal keratitis. A history of previous OSD and ocular surgery was more frequently observed in the PS group. These findings were likely due to the past use of steroids prescribed for treatment of the patients' underlying conditions. A previous Korean study reported that in 14.1% of fungal keratitis cases, patients had been prescribed topical steroids prior to their diagnosis [10]. Studies in other countries have reported a range from 13% to 44% [11–13].

The types and distribution of the microbiological profile of fungal keratitis vary according to geography, climate, and the socioeconomic characteristics of the patients involved. In this study, the most commonly identified organism in both groups was *Candida* species (20% in PS and 11.3% in NPS) followed by *Fusarium* species. Our findings in relation to *Candida* species are similar to the results of studies conducted across the globe, i.e., London (60.6%) [14], Paris (58%) [15], Denmark (52%) [11], and Pennsylvania (45.8%) [16]. In contrast, studies in north China (73.3%) [17], Florida (41%) [18], Mexico City (37.2%) [19], south India (37.2%) [20], central China (30.6%) [21], and Korea (29%) [10] reported that *Fusarium* was the most commonly identified organism. *Aspergillus* was the most commonly identified organism in reports from north India (41%) and Saudi Arabia (27.2%) [22, 23]. Our study determined that *Aspergillus* was found only in the PS group. This result can be supported by the study of Tony et al. who had reported that corticosteroids promote the growth of *Aspergillus* [24].

The authors anticipated that the PS group would have more severe initial clinical characteristics than the NPS group. However, our study found no significant differences in initial clinical characteristics between the two groups except in terms of depth of infiltration. We speculate that this finding may be related to the inflammation-masking effect of previous topical steroids used in early stage of keratitis. This finding may also make clinical suspicion and early diagnosis of fungal keratitis difficult. At initial presentation, deep infiltration of the infection was higher in the PS group. In a study by Panda et al., it was reported that hyphae are located more vertically in a group that used steroids [25]. Fungi are characterized by deep penetration into the corneal stroma, and vertically located hyphae are more involved in penetration and are more virulent [25]. A study by Lixin et al., found that the vertically growing hyphae had a higher recurrence rate after lamellar keratoplasty than horizontally growing hyphae [26]. Therefore, it is important to evaluate the detailed characteristics of the lesion and to collect a comprehensive patient history at the initial visit.

In this study, only microbiologically proven fungal keratitis was included, and microbiological evidences of fungus were made through potassium hydroxide smear, culture, PCR, or biopsy. The percentage of identified fungal isolates was higher in the PS group than in the NPS group. One potential interpretation of this result is that steroid use can promote fungal proliferation, thereby enhancing its identification. However, the relationship between the use of prior topical steroids and the positive rate of culture has rarely been reported. Further studies are needed to investigate the origin of relationship. Furthermore, this study does not have a prospective design, and it does not include the cases of negative microbiological tests. Therefore, there is a limit to evaluation and interpretation.

There was no significant difference in the type and proportion of antifungal agents used between the two groups, but topical and systemic voriconazole use was significantly higher in the PS group. In the medical center where the authors practice, the use of topical and systemic voriconazole when there is no response to conventional antifungal therapy. The significantly higher use of topical and systemic voriconazole in the PS group indicates that the treatment response was worse than that anticipated in this group.

The PS group had significantly higher surgical intervention and treatment failure than the NPS group. This is consistent with the results of other studies that have suggested that the prior use of topical steroids in fungal keratitis may contribute to worse outcomes [27, 28]. These results highlight the side effects of prior topical steroid use in the cases of fungal keratitis. Evisceration/enucleation was performed in 13.3% of the overall patients. These results were similar to the proportion of eviceration/enucleation (10.6%) reported in a multicenter study in Korea [10]. The authors expected that there would be higher incidence of evisceration/enucleation in the PS, just as more surgical treatments were needed in the PS group. However, in this study, no significant difference was observed in the proportion of evisceration/enucleation between the PS and the NPS groups (13.3%, 13.2%). Therefore, we performed an additional logistic regression analysis to determine the risk factors for evisceration/enucleation. As a result, hypopyon (PS: 30%, NPS: 32%) was the only significant risk factor for evisceration/enucleation (OR 4.88, 95% CI 1.28–18.56, *p* = 0.020). Thus, the risk factors for evisceration/enucleation was associated with initial clinical severity of the overall cohort, and further study will be needed.

In this study, significant risk factors for treatment failure were hypopyon and deep infiltration. Prior topical steroid use and previous OSD were significant in univariate logistic regression analysis but their effects were attenuated in multivariate analysis. Hypopyon can be regarded as a marker of inflammation. A study of Lalitha et al. reported that the presence of hypopyon was a significant predictor of treatment failure [29]. And deep stromal infiltration was a significant risk factor for treatment failure. The depth of infiltration may reflect the progression of the lesion, and the poor corneal penetration of antifungal agents may be related to the difficulty of treatment in the case of deep infiltration [3, 4]. Therefore, it is important to evaluate these features at the initial visit because fungal keratitis can penetrate deeply into the stroma in the early stage of infection. Other studies have reported various risk factors for treatment failure in fungal keratitis such as large epithelial defect size and prior topical steroid use [30, 31].

The reported role of steroids in fungal keratitis includes suppression of inflammation and subsequent growth promotion of the fungal genus. Moreover, vertically oriented hyphae are more commonly observed in the eyes of patients who used steroids [25]. Steroid use has been associated with a decreased response to antifungal agents, and steroid treatment itself is a known risk factor for fungal infection [8, 28, 32]. Steroid worsen infections due to severe inflammatory side effects and they also affect the delay of epithelial regeneration [33–36]. Therefore, it should be emphasized that early steroid use is contraindicated when an infection is suspected. Clinicians should be cautious when prescribing steroids for suspected cases of infectious keratitis.

This study has some limitations. First, this study was confined to South Korea, which is temperate climate. And the cases included were from one tertiary hospital. Therefore, the results of this study cannot be generalized. Second, owing to the study’s retrospective design, the authors could not accurately identify the potency and dose of the topical steroids prescribed for patients who were referred from their primary eye clinics. Third, only the patients with microbiological evidence of fungal keratitis were enrolled in this study while cases without such evidence were excluded, even if fungal keratitis was highly suspected. Despite such limitations, this study has an important clinical significance. This investigation highlighting the risk and side effects associated with prior topical steroid use in practical clinical circumstances. This study is a clinical analysis of fungal keratitis in South Korea. Clinician may use these findings as a beneficial reference for various regional differences in fungal keratitis.

## Conclusions

In this study, 36.1% of the patients used topical steroids prior to their diagnosis of microbiologically proven fungal keratitis. Previous OSD and deep infiltration were more common in the PS group than the NPS group. The use of systemic or topical voriconazole, surgical intervention and treatment failure were more common in the PS group. Hypopyon and deep infiltration of the infection were significant risk factors for treatment failure. Considering the risk for complications, the results of this study suggest that topical steroids should be administered with caution. Therefore, it is important to use topical steroids judiciously in ocular surface diseases such as keratitis, and to monitor patients frequently during steroid treatment.

## Data Availability

The datasets used and/or analyzed during the current study available from the corresponding author on reasonable request.

## Abbreviations

BCVA: Best-corrected visual acuity
CI: Confidence interval
EHT: Epithelial healing time
KOH: potassium hydroxide
NPS: No prior topical steroid use group
OR: Odds ratio
OSD: Ocular surface disease
PCR: Polymerase chain reaction
PS: Prior topical steroid use group
